# Fibroblast Growth Factor 2 Protein Stability Provides Decreased Dependence on Heparin for Induction of FGFR Signaling and Alters ERK Signaling Dynamics

**DOI:** 10.3389/fcell.2019.00331

**Published:** 2019-12-12

**Authors:** Zuzana Koledova, Jakub Sumbal, Anas Rabata, Gabin de La Bourdonnaye, Radka Chaloupkova, Barbara Hrdlickova, Jiri Damborsky, Veronika Stepankova

**Affiliations:** ^1^Department of Histology and Embryology, Faculty of Medicine, Masaryk University, Brno, Czechia; ^2^International Clinical Research Center, St. Anne’s University Hospital, Brno, Czechia; ^3^Enantis, Brno, Czechia; ^4^Loschmidt Laboratories, RECETOX and Department of Experimental Biology, Faculty of Science, Masaryk University, Brno, Czechia

**Keywords:** extracellular-signal-regulated kinase (ERK), fibroblasts, fibroblast growth factor, fibroblast growth factor receptor, heparin, primary fibroblasts, signaling

## Abstract

Fibroblast growth factor 2 (FGF2) plays important roles in tissue development and repair. Using heparan sulfates (HS)/heparin as a cofactor, FGF2 binds to FGF receptor (FGFR) and induces downstream signaling pathways, such as ERK pathway, that regulate cellular behavior. In most cell lines, FGF2 signaling displays biphasic dose-response profile, reaching maximal response to intermediate concentrations, but weak response to high levels of FGF2. Recent reports demonstrated that the biphasic cellular response results from competition between binding of FGF2 to HS and FGFR that impinge upon ERK signaling dynamics. However, the role of HS/heparin in FGF signaling has been controversial. Several studies suggested that heparin is not required for FGF-FGFR complex formation and that the main role of heparin is to protect FGF from degradation. In this study, we investigated the relationship between FGF2 stability, heparin dependence and ERK signaling dynamics using FGF2 variants with increased thermal stability (FGF2-STABs). FGF2-STABs showed higher efficiency in induction of FGFR-mediated proliferation, lower affinity to heparin and were less dependent on heparin than wild-type FGF2 (FGF2-wt) for induction of FGFR-mediated mitogenic response. Interestingly, in primary mammary fibroblasts, FGF2-wt displayed a sigmoidal dose-response profile, while FGF2-STABs showed a biphasic response. Moreover, at low concentrations, FGF2-STABs induced ERK signaling more potently and displayed a faster dynamics of full ERK activation and higher amplitudes of ERK signaling than FGF2-wt. Our results suggest that FGF2 stability and heparin dependence are important factors in FGF-FGFR signaling complex assembly and ERK signaling dynamics.

## Introduction

Fibroblast growth factor 2 (FGF2) is a canonical member of the fibroblast growth factor family that exerts multiple functions in tissue development and repair through binding to fibroblast growth factor receptors 1, 2 and 3 (FGFR1-3) (Ornitz and Itoh, 2015). Of the FGFR isoforms “b” (IIIb) and “c” (IIIc) that are generated by alternative splicing of the extracellular immunoglobulin domain III, FGF2 preferentially interacts with splice variants FGFR1c, FGFR2c, FGFR3c, and FGFR1b (Ornitz et al., 1996). FGF2 binding to FGFR results in FGFR dimerization and transphosphorylation of its tyrosine kinase domain (Spivak-Kroizman et al., 1994; Ornitz et al., 1996). FGFR activation induces intracellular signaling pathways, including RAS-MAPK (ERK1/2), PI3K-AKT, PLCγ-PKC, and STAT (Ornitz and Itoh, 2015).

Heparan sulfates, including heparin, act as co-receptors of FGF2. Early studies suggested that HS/heparin is directly involved in FGFR dimerization and required for efficient FGFR activation and induction of FGF-mediated mitogenic response (Yayon et al., 1991; Ornitz et al., 1992; Waksman and Herr, 1998; Lundin et al., 2003; Wu et al., 2003; Mohammadi et al., 2005). However, several contradictory studies emerged, reporting that FGF can interact with FGFR and trigger activation of downstream signaling pathways, including ERK1/2, even in the absence of HS (Nugent and Edelman, 1992; Roghani et al., 1994; Fannon and Nugent, 1996; Delehedde et al., 2000, 2002). But the activation of signaling pathways in the absence of HS was only transient and inefficient to promote mitogenic response (Delehedde et al., 2002; Zhu et al., 2010). Sustained ERK1/2 signaling that elicited cell proliferation was found dependent on HS and FGF2 concentration (Zhu et al., 2010).

Besides its role in mediating FGF-FGFR interaction and complex formation, HS/heparin protects FGFs from inactivation *in vivo*. FGF2 is susceptible to aggregation, heat, acidic pH and proteolytic degradation, leading to the loss of its biological activity and function and to short half-life (≤10 h at 37°C) (Dvorak et al., 2018). Binding to HS/heparin increases FGF2 stability (Gospodarowicz and Cheng, 1986; Caldwell et al., 2004), increasing the denaturation temperature of FGF2 by more than 20°C (Buchtova et al., 2015). An elegant study on FGF1, another unstable FGF protein, that used FGF1 variants with reduced affinity for heparin and with diverse stability, suggested that stabilization of FGF1 is the main role of heparin in FGF-induced signaling and that heparin is not essential for a direct FGF1-FGFR interaction and FGFR activation (Zakrzewska et al., 2009). However, the relationship between FGF2 stability, dependence on heparin for FGFR signaling and the resulting ERK1/2 signaling dynamics have not been fully elucidated.

In this study, we used FGF2 variants with increased thermal stability, FGF2-STAB1 and FGF2-STAB2, created by computer-assisted protein engineering (Dvorak et al., 2018) to investigate the effect of FGF2 stability on FGF2-induced cell proliferation, dependence on heparin, and ERK1/2 signaling dynamics. After characterization of FGFR specificity and thorough testing of thermal stability of FGF2-STABs, we report on results from testing of heparin requirement for induction of proliferation in response to FGF2-wt and FGF2-STABs in BaF3-FGFR cell lines. We also present our findings from studies of ERK1/2 phosphorylation dynamics in primary mammary fibroblasts.

## Materials and Methods

### Preparation of FGF2 Protein Variants

To overproduce FGF2-wt, FGF2-STAB1 and FGF2-STAB2 in *Escherichia coli*, the corresponding genes *fgf2-G0*, *fgf2-G2*, and *fgf2-G3*, respectively (Dvorak et al., 2018), were expressed under the control of the T7*lac* promoter and the gene expression was induced by the addition of isopropyl β-D-thiogalactopyranoside (IPTG). *E. coli* BL21(DE3) cells containing recombinant plasmids pET28b-His-thrombin:*fgf2-wt*, pET28b-His-thrombin:*fgf2-STAB1* and pET28b-His-thrombin:*fgf2-STAB2* were grown in 1 l of Luria broth medium with 50 μg/ml kanamycin at 37°C. When the culture reached an optical density 0.6 at 600 nm, the induction of protein expression (at 20°C) was initiated by the addition of IPTG to a final concentration of 0.5 mM. The cells were harvested, disrupted by sonication and centrifuged for 1 h at 4°C and 21,000 × *g*. FGF2 variants were purified on cOmplete His-Tag Purification Columns (Merck) attached to ÄKTA FPLC purification system (GE Healthcare). His-tagged proteins were bound to the resin in equilibrating buffer (20 mM KH_2_PO_4_, pH 7.5; 0.5 M NaCl, 10 mM imidazole). Unbound and weakly bound proteins were washed out. His-tagged proteins were eluted by a linear gradient (0 – 100%) of purification buffer containing 500 mM imidazole. The eluted proteins were pooled and dialyzed overnight against 20 mM phosphate buffer (16.4 mM K_2_HPO_4_, 3.6 mM KH_2_PO_4_), pH 7.5, with 750 mM NaCl. Protein concentration was determined using Bradford reagent (Merck) and protein purity was checked by SDS-PAGE. Then the proteins were lyophilized at 1 mg/ml using laboratory freeze-dryer Alpha 1–2 LD plus (Martin Christ) and stored at 4°C. The lyophilized proteins were reconstituted in ice cold PBS at 500 μg/ml, sterile filtered through 0.22 μm filter and protein concentration of the resulting solution was measured using Bradford reagent. The protein solution was further diluted to 100 μg/ml FGF2 in PBS with 0.1% bovine serum albumin (Merck), aliquoted and stored at −20°C. For each experiment, freshly ice-thawed aliquots were used. Five different batches of FGF2-wt, FGF2-STAB1 and FGF2-STAB2 were used in this study.

### BaF3 Cell Culture and Proliferation Assay

BaF3 cells expressing FGFR isotypes (Ornitz et al., 1996) were maintained in RPMI-1640 medium (Biosera) with 10% newborn calf serum (NCS; Merck), 4 mM L-glutamine, 100 U/ml of penicillin, 100 μg/ml of streptomycin (i.e., 1 × Pen/Strep) (all Thermo Fisher Scientific), 600 μg/ml G418, 50 μM β-mercaptoethanol (both Merck) and 0.5 ng/ml mouse interleukin 3 (IL3; Peprotech).

For BaF3 proliferation assays, 4 × 10^4^ cells per well were seeded in 96-well plates in BaF3 basal medium [with serum: 10% NCS, 1 × Pen/Strep in RPMI-1640; or serum-free: 0.05 × insulin-transferrin-selenium (ITS; Thermo Fisher Scientific), 1 × Pen/Strep in RPMI-1640] with or without heparin (0–2 μg/ml; Merck), or with protamine sulfate (250 μg/ml; Merck) as required for the experiments and incubated overnight at 37°C, 5% CO_2_. The next day the FGF2 variants at different concentrations (as needed for the experiment) were added to the plates. Within each experiment, all treatments were done in triplicates. The cells were incubated with FGF2 variants for 1–4 days (24 h for testing of heparin dependence in medium with serum; 2 days for FGFR specificity testing, testing of heparin dependence in serum-free conditions, and tests with protamine sulfate; 4 days for thermostability testing). Then resazurin (Merck) was added to the plate to the final concentration 10 μg/ml and the plates were incubated for 6–24 h (until resazurin color change was observed). The same incubation time was strictly adhered to for all plates within the same experiment. Resorufin fluorescence (excitation at 560 nm, emission at 590 nm) was measured using Synergy H4 Hybrid multi-mode microplate reader (BioTek). EC_50_ values were calculated from normalized data using non-linear regression in GraphPad Prism.

### Differential Scanning Fluorimetry

Freshly prepared FGF2 variants were mixed with heparin (Merck) at different ratios to a final concentration of 1 mg/ml FGF2 and 0–12 μM heparin (in 20 mM phosphate buffer, pH 7.5, with 750 mM NaCl). Standard grade capillary (NanoTemper) was filled with a sample and placed into the Prometheus NT.48 (NanoTemper). The samples were continually heated from 20 to 90°C at scan rate 1°C/min and fluorescence signal excited at 295 nm with an excitation power of 70% was followed at 330 and 350 nm. Unfolding transition points were determined from changes in the emission wavelengths of tryptophan fluorescence at 330 nm, 350 nm, and their ratios.

### Isothermal Titration Calorimetry

FGF2 variants were dialyzed against a reaction buffer (20 mM phosphate buffer, pH 7.5, with 750 mM NaCl) and degassed for 10 min in the Thermo Vac (MicroCal) prior to use. The measurements were performed on a VP-ITC isothermal titration calorimeter (MicroCal) at 25°C. Protein solutions (1.2 – 2.1 mg/ml) were titrated by 7 μl aliquots of heparin solution (250 μM in the reaction buffer) with 7 min spacing between each titrant addition to assure signal returning baseline. The titrant was added until saturation was observed. The maximum total number of heparin additions was 40. The integrated heat changes were then plotted against the molar ratio and analyzed with Origin^®^ scientific plotting software version 7.0 (MicroCal) using a One Set of Sites curve fitting model to obtain the association constant (*K*_a_), the stoichiometry, the enthalpy as well as the entropy of binding. The *K*_d_ of the binding was calculated as *K*_d_ = 1/*K*_a_.

### Primary Mammary Fibroblast Isolation and Culture

Primary mammary fibroblasts were isolated from 6–8 weeks old ICR mice as previously described (Koledova, 2017). The animals were obtained from the Laboratory Animal Breeding and Experimental Facility of the Faculty of Medicine, Masaryk University. Experiments involving animals were approved by the Ministry of Agriculture of the Czech Republic, supervised by the Expert Committee for Laboratory Animal Welfare at the Faculty of Medicine, Masaryk University, and performed by certified individuals (ZK, JS). The study was carried out in accordance with the principles of the Basel Declaration. Primary mammary fibroblasts were cultured in fibroblast cultivation medium [DMEM (Thermo Fisher Scientific), 10% FBS (HyClone), 1 × ITS, 1 × Pen/Strep] and used for the experiments until passage 5.

### Analysis of ERK Signaling Dynamics

Fibroblasts were serum-starved by culture in DMEM with 0.05 × ITS, 1 × Pen/Strep for 24 h and then treated with no FGF (mock) or with 0.001–150 nM of FGF2 variants in DMEM, with or without heparin (4 μg/ml), for 5–60 min. Then the cells were washed with ice-cold PBS and lysed in RIPA buffer [150 mM NaCl, 1.0% NP-40, 0.5% sodium deoxycholate, 0.1% SDS, 50 mM Tris, pH 8.0] supplied with proteinase and phosphatase inhibitors (10 mM β-glycero-phosphate, 5 mM NaF, 1 mM Na_3_VO_4_, 1 mM dithiotreitol, 0.5 mM phenylmethanesulphonyl fluoride, 2 μg/ml aprotinin, 10 μg/ml leupeptin; all Merck).

BaF3 cells were IL3-starved by culture in BaF3 basal medium (with 10% NCS and 600 μg/ml G418) for 24 h, and then treated with no FGF (mock) or with 0.1–10 nM of FGF2 variants in BaF3 basal medium, with or without heparin (2 μg/ml), for 5–60 min. The cells were immediately placed on ice and treated with phosphatase inhibitors (5 mM NaF, 0.2 mM Na_3_VO_4_), pelleted by centrifugation at 4°C and washed with ice-cold PBS with phosphatase inhibitors (5 mM NaF, 0.2 mM Na_3_VO_4_). The cells were pelleted by centrifugation and lysed in RIPA supplied with proteinase and phosphatase inhibitors.

### Western Blotting

Protein lysates were homogenized by vortexing, cleared by centrifugation and protein concentration was measured using the Bradford reagent. Denatured, reduced samples were resolved on 10% SDS-PAGE gels and blotted onto PVDF membranes. Membranes were blocked with 5% non-fat milk in PBS (blocking buffer) with 0.05% Tween-20 (Merck) and incubated with primary antibodies diluted in blocking buffer (1:1000) overnight at 4°C. After washing in PBS with 0.05% Tween-20, membranes were incubated with horseradish peroxidase-conjugated secondary antibodies diluted in 5% milk (1:2000) for 1 h at room temperature. Signal was developed using a chemiluminiscence substrate (100 mM Tris-HCl, pH 8.5, 0.2 mM coumaric acid, 1.25 mM luminol, 0.01% H_2_O_2_) and exposed on X-ray films (Agfa), which were then scanned and band density was analyzed using Western Blot densitometry analysis - macro tool for ImageJ 1.x (Cernek, 2019). Phosphorylated and total proteins and actin/tubulin were analyzed on a single blot. Primary antibodies used: β-actin (Merck, #A1978; Santa Cruz Biotechnology, sc-47778); P-ERK1/2 (Thr202/Tyr204) (Santa Cruz Biotechnology, sc-16982-R; Cell Signaling Technology, #4370), α-tubulin (Santa Cruz Biotechnology, sc-8035); ERK1/2 (Cell Signaling Technology, #9102). Secondary antibodies used: anti-mouse antibody and anti-rabbit antibody (Cell Signaling Technology).

### Data Analysis and Presentation

Statistical analysis and EC_50_ calculation were performed using GraphPad Prism software. Line plots and bar graphs were generated by GraphPad Prism and show mean ± standard deviation (SD) or standard error of mean (SEM). The number of independent biological replicates is indicated as *n*. The *P*-values are indicated as ^∗^*P* < 0.05; ^∗∗^*P* < 0.01; ^∗∗∗^*P* < 0.001; ^****^P < 0.0001; n.s., not significant.

Results from multiple statistical analyses of complex data sets are presented in color maps. The color maps are association matrices that show results from a pairwise comparison of all pairs of experimental variants within an experiment in a table-like form. The experimental variants are listed in line headings vertically and, in the same order but horizontally, in column headings, making the matrix symmetric by diagonal. *P*-value summary for each and any pair of compared experimental variants is indicated (by asterisks as well as color code) in the box at the crossing of the respective line for one of the variants and the column for the other variant. The diagonal consists of crossings of the lines and columns of the same experimental variants, where statistical comparison is not applicable (NA).

## Results

### FGF2-STABs Have the Same FGFR Specificity as FGF2-wt and Show Increased Efficiency at Inducing FGFR-Mediated Cell Response

First we tested the FGFR specificity of FGF2-STABs using the BaF3-FGFR cell model system. BaF3 cells do not naturally express HS or FGFRs, and their proliferation is IL3 dependent. Stable transgenic BaF3 cell lines carrying six major splice variants of FGFRs (FGFR1b, FGFR1c, FGFR2b, FGFR2c, FGFR3b, and FGFR3c) enable testing of FGF binding to FGFR (Ornitz et al., 1996). Upon depletion of IL3, BaF3-FGFR lines become FGF-dependent for induction of proliferation. Therefore, cell proliferation assays, such as resazurin assay, can be used as a direct indicator of FGF binding to the FGFR in respective BaF3 lines.

**FIGURE 1:**
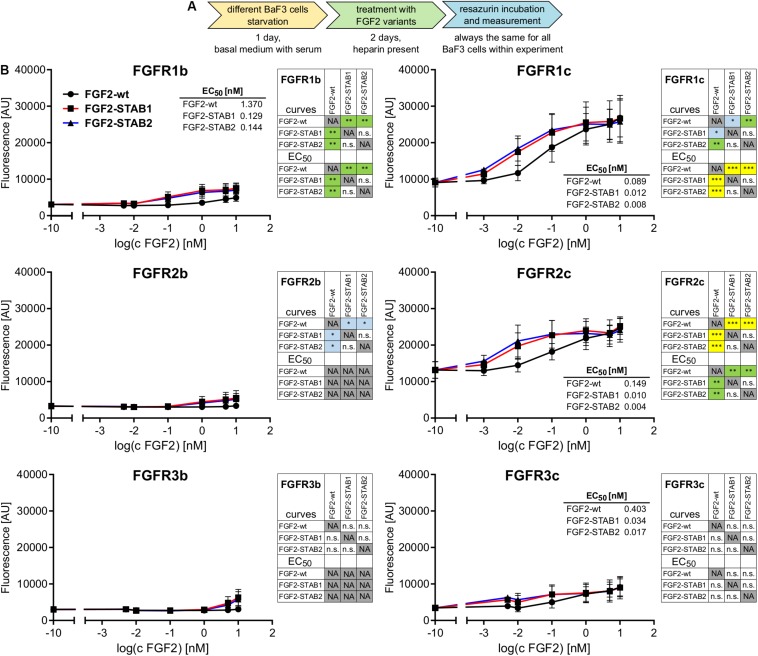
FGF2-STABs have the same FGFR specificity as FGF2-wt. **(A,B)** FGFR specificity testing using BaF3 proliferation assay. **(A)** Experimental design scheme. BaF3-FGFR cells were seeded in basal medium containing serum and treated with FGF2 variants in the presence of heparin (2 μg/ml) for 2 days. **(B)** The line plots show resorufin fluorescence, measured after 2 days of culture with FGF2 variants, as mean ± SD; *n* = 2 for FGFR1b, FGFR2b, FGFR3b, FGFR3c; *n* = 8 for FGFR1c, FGFR2c. The insets show EC_50_ values of FGF2 variants for respective FGFR. The color maps show results of statistical comparison of whole curves (two-way ANOVA) and EC_50_ values (one-way ANOVA, Tukey’s multiple comparisons test). ^∗^*P* < 0.05; ^∗∗^*P* < 0.01; ^∗∗∗^*P* < 0.001; n.s., not significant. NA, not applicable.

To this end, BaF3-FGFR cells were treated with FGF2 variants (in the presence of heparin) for 2 days (Figure 1A). Treatment of BaF3-FGFR cells with FGF2-wt induced proliferation in cells expressing FGFR1c, FGFR2c, FGFR3c, or FGFR1b (Figure 1B), which is consistent with published data (Ornitz et al., 1996; Ornitz and Itoh, 2015). Similarly, FGF2-STAB1 and FGF2-STAB2 effectively induced proliferation of BaF3-FGFR1c, -FGFR2c, -FGFR3c, and -FGFR1b cells and did not significantly induce proliferation of BaF3-FGFR2b or -FGFR3b cells (Figure 1B). Importantly, comparison of half maximal effective concentrations (EC_50_) revealed that FGF2-STABs were more effective in inducing BaF3 cell proliferation than FGF2-wt. In general, the EC_50_ values of FGF2-STABs were about 10-fold to 100-fold lower than the EC_50_ values of FGF2-wt (Figure 1B), suggesting that increasing FGF2 stability increased FGF2 affinity for FGFRs.

### FGF2-STABs Show Increased Thermostability That Is Independent on Heparin

**FIGURE 2:**
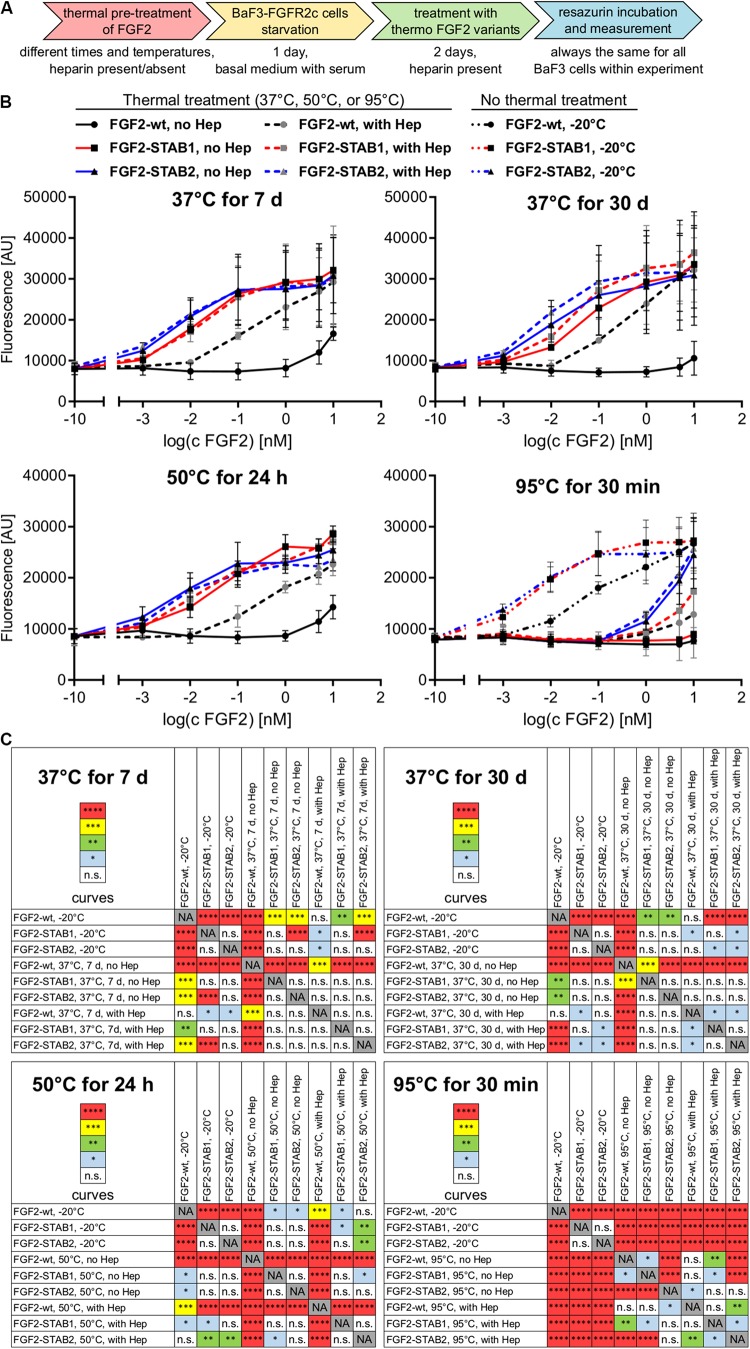
FGF2-STABs show increased thermal stability. **(A–C)** Thermostability testing using proliferation assay of BaF3-FGFR2c cells. **(A)** Experimental design scheme. FGF2 variants were exposed to 37, 50, or 95°C in the presence (2 μg/ml heparin; with Hep) or absence of heparin (no Hep) for the times indicated, or not thermally treated at all but stored at −20°C, and then used to treat the BaF3-FGFR2c cells. BaF3-FGFR2c cells were seeded in basal medium containing serum and treated with FGF2 variants in the presence of heparin (2 μg/ml) for 4 days. **(B)** The line plots show resorufin fluorescence, measured after 4 days of culture with FGF2 variants, as mean ± SD, *n* = 2–3. For visual clarity, the plots for thermally non-treated FGF2 variants (−20°C) are shown only in the plots with 95°C-treated variants, otherwise they were too much overlapping with the curves in graphs of other thermal treatments. **(C)** The color maps show results of statistical comparison of whole curves of FGF2 variants after thermal treatment. ^∗^*P* < 0.05; ^∗∗^*P* < 0.01; ^∗∗∗^*P* < 0.001; ^****^P < 0.0001; n.s., not significant (two-way ANOVA).

Next, we thoroughly tested the thermal stability of FGF2-STABs. FGF2-wt, FGF2-STAB1 and FGF2-STAB2 were incubated at 37°C for 7 days or 30 days, at 50°C for 24 h, or at 95°C for 30 min at the concentration 10 μg/ml in the absence or presence of heparin (2 μg/ml), or not thermally treated at all (stored at −20°C) and then used in BaF3-FGFR1c and BaF3-FGFR2c proliferation assays (Figure 2A). These two cell lines were selected for the test because they were the most responsive to FGF2 as revealed by the test for FGFR specificity (Figure 1B). To test the activity of FGF2 variants after thermal treatments, BaF3-FGFR2c and BaF3-FGFR1c cells were exposed to FGF2 variants (in the presence of heparin) for 4 days.

**FIGURE 3:**
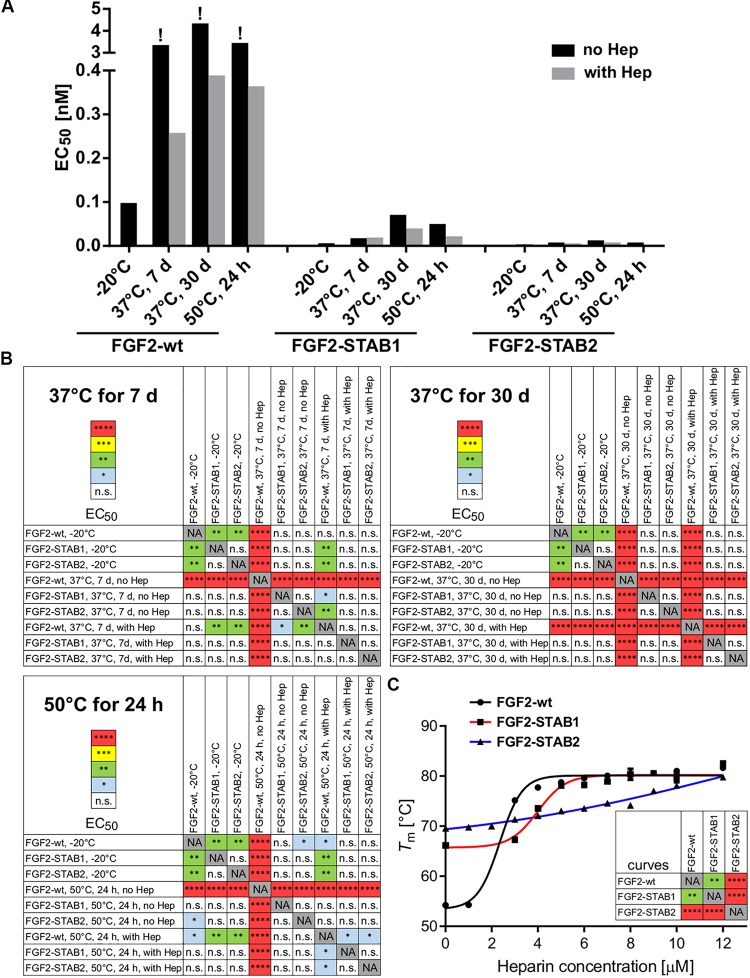
FGF2-STABs show increased thermal stability, as indicated by their lower EC_50_ values. **(A)** The bar plot presents EC_50_ values of FGF2 variants after thermal treatments, calculated from data presented in Figure 2A. The exclamation mark (!) indicates EC_50_ values that are only rough estimates from the available data; however, true EC50 values are most likely higher because at the range of concentrations tested, maximum response was not reached. **(B)** The color maps show results of statistical analysis of EC_50_ values of FGF2 variants after thermal treatment, calculated from data presented in Figure 2A. ^∗^*P* < 0.05; ^∗∗^*P* < 0.01; ^∗∗∗^*P* < 0.001; ^****^P < 0.0001; n.s., not significant (one-way ANOVA, Tukey’s multiple comparisons test). NA, not applicable. **(C)** Dependence of the melting temperature (*T*_m_) of the FGF2 variants on heparin concentration. The data were fitted to sigmoidal curves. The color maps show results of statistical comparison of whole curves (two-way ANOVA). ^∗∗^*P* < 0.01; ^****^P < 0.0001. NA, not applicable.

Treatment of FGF2-wt at 37°C for 7 days in the absence of heparin led to a significant decrease in FGF2 activity, which was revealed by a significant, almost 23-fold increase of EC_50_ in BaF3-FGFR2c cells. Presence of heparin during the thermal treatment stabilized FGF2-wt and the EC_50_ increased only 1.7-fold. Incubation of FGF2-wt at 37°C for 30 days or at 50°C for 24 h in the absence of heparin increased the EC_50_ 29- and 23-fold, respectively. Presence of heparin stabilized FGF2-wt and EC_50_ increased only 2.6- and 2.5-fold, respectively (Figures 2B,C, 3A,B and Table 1).

**TABLE 1:** The dependence of EC_50_ values of FGF2 variants on thermal treatment and heparin.

**FGF2 variant**	**Thermal treatment**	**EC_50_ [nM]**	
		**No heparin**	**With heparin**
FGF2-wt	−20°C	0.098	NA
	37°C, 7 days	3.360 (!)	0.258
	37°C, 30 days	4.342 (!)	0.389
	50°C, 24 h	3.450 (!)	0.364
FGF2-STAB1	−20°C	0.006	NA
	37°C, 7 days	0.018	0.019
	37°C, 30 days	0.071	0.040
	50°C, 24 h	0.050	0.022
FGF2-STAB2	−20°C	0.004	NA
	37°C, 7 days	0.008	0.006
	37°C, 30 days	0.013	0.008
	50°C, 24 h	0.008	0.007

*The EC_50_ values were calculated from experiments in BaF3-FGFR2c cells from plots depicted in Figure 1B. The exclamation mark (!) indicates rough estimate values from the available data; however, true EC_50_ values are most likely higher because at the range of concentrations tested, maximum response was not reached.*

FGF2-STABs showed a minor to a negligible decrease in activity after thermal treatment at 37 and 50°C (Figures 2B,C, 3A,B and Table 1). After incubation at 37°C for 7 days in the absence of heparin, the EC_50_ increased 1.9- and 2-fold for FGF2-STAB1 and FGF2-STAB2, respectively, and the presence of heparin had a minor effect on FGF2-STAB1 or FGF2-STAB2 stability. Incubation of FGF2-STABs at 37°C for 30 days or at 50°C for 24 h in the absence of heparin slightly increased the EC_50_ values but they were still at least an order of magnitude lower than the EC_50_ of FGF2-wt stored at −20°C. In the presence of heparin, the EC_50_ increased only 4.2- and 2.3-fold for FGF2-STAB1, and 2.1- and 1.8-fold for FGF2-STAB2 at 37°C (30 days) and 50°C (24 h), respectively (Figures 2B,C, 3A,B and Table 1). Similar results were obtained from tests in BaF3-FGFR1c cells (Supplementary Figures 1A–C, 2A,B and Supplementary Table 1). In sum, according to both the lowest absolute values of EC_50_ and to the smallest fold changes of EC_50_ values after thermal treatment, FGF2-STAB2 was the most stable variant from all the FGF2 variants tested. Interestingly, FGF2-STAB2 retained some activity also after treatment at 95°C for 30 min (Figures 2B,C and Supplementary Figures 1B,C), independently on heparin.

To determine the dependence of thermal stability of FGF2 variants on heparin, *T*_m_ of the FGF2 variants at a range of heparin concentrations was measured using differential scanning fluorimetry. The highest *T*_m_ (≈80°C), corresponding to the highest thermostability, was achieved at the highest concentration of heparin tested (12 μM) for all FGF2 variants (Figure 3C). However, the contribution of heparin to FGF2 stabilization was different for individual FGF2 variants. FGF2-wt was stabilized by 27°C (Δ*T*_m_) in the presence of 12 μM heparin, while FGF2-STAB1 and FGF2-STAB2 variants were stabilized by only 12 and 10°C, respectively (Figure 3C). At the same time, the transition curve of heparin-mediated stabilization of FGF2-STABs was much wider than that of FGF2-wt. The inflection points of the sigmoidal dependence, corresponding to the heparin concentration, at which the protein reaches half maximal stabilization (SC_50_), were 2.4, 4.3, and 6.4 μM for FGF2-wt, FGF2-STAB1 and FGF2-STAB2, respectively (Figure 3C). In conclusion, the smaller Δ*T*_m_ and higher SC_50_ of FGF2-STABs in comparison to FGF2-wt demonstrated increased independence of FGF2-STAB1 and FGF2-STAB2 proteins on heparin for stabilization. This might be due to the inherent stability of FGF2-STABs being so high that heparin can contribute only a very small stabilization effect.

### FGF2-STABs Are Less Dependent on Heparin for Induction of FGFR Signaling Than FGF2-wt

**FIGURE 4:**
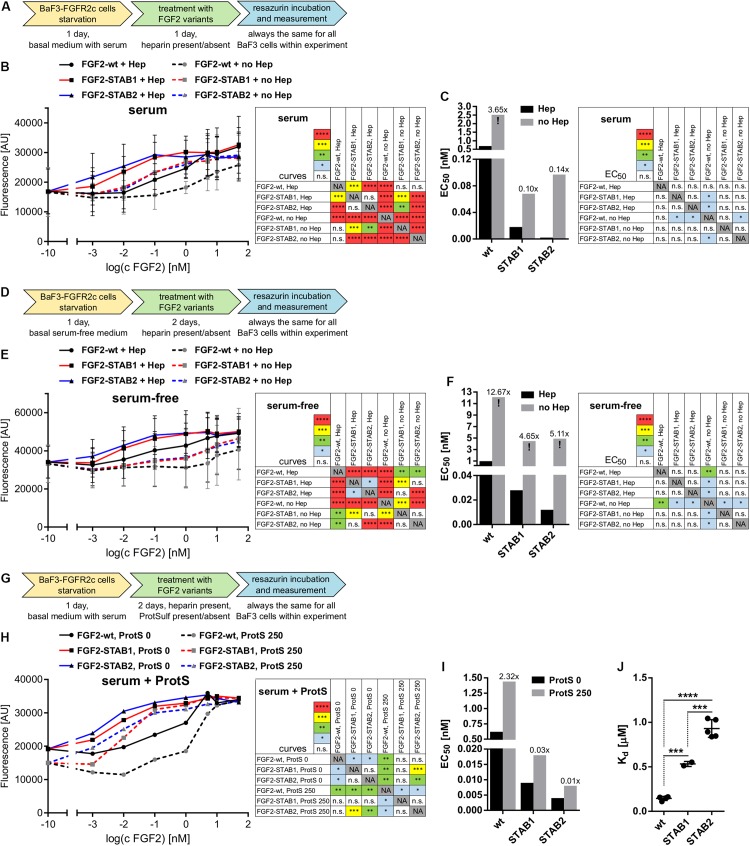
FGF2-STABs are less dependent on heparin for induction of FGF signaling. **(A–I)** Proliferation assays on BaF3-FGFR2c cells. **(A–C)** Testing the ability of FGF2 variants to induce FGFR signaling with or without the addition of heparin in medium with serum. **(A)** Experimental design scheme. BaF3-FGFR2c cells were seeded in basal medium containing serum and treated with FGF2 variants in the presence (2 μg/ml heparin; with Hep) or absence of heparin (no Hep) for 24 h. **(B)** The line graph shows resorufin fluorescence as mean ± SD, *n* = 2. **(C)** The bar plot shows EC_50_ of FGF2 variants as calculated from the line plot in **(B)**. The numbers above the bars indicate fold increase of the respective EC_50_ value in comparison to EC_50_ of FGF2-wt in the presence of heparin (EC_50_:FGF2-wt + Hep). **(D–F)** Testing the ability of FGF2 variants to induce FGFR signaling with or without the addition of heparin in serum-free medium. **(D)** Experimental design scheme. The cells were seeded in serum-free basal medium and treated with FGF2 variants in the presence (2 μg/ml heparin; with Hep) or absence of heparin (no Hep) for 48 h. **(E)** The line graph shows resorufin fluorescence as mean ± SD, *n* = 2. **(F)** The bar plot shows EC_50_ of FGF2 variants as calculated from the line plot in **(E)**. The numbers above the bars indicate fold increase of the EC_50_ value in comparison to EC_50_:FGF2-wt + Hep. **(G–I)** Testing the requirement of heparin for induction of FGF signaling by FGF2 variants using protamine sulfate, an inhibitor of heparin. **(G)** Experimental design scheme. The cells were seeded in basal medium containing serum and treated with FGF2 variants in the presence of heparin (2 μg/ml) and 0 or 250 μg/ml protamine sulfate (ProtS) for 48 h. **(H)** The line graph shows resorufin fluorescence, *n* = 1. **(I)** The bar plot shows EC_50_ of FGF2 variants calculated from the plot **(H)**. The numbers above the bars indicate fold increase of the EC_50_ value in comparison to EC_50_:FGF2-wt + Hep (without ProtS). The color maps show results of statistical comparison of whole curves (two-way ANOVA) and EC_50_ values (one-way ANOVA, Tukey’s multiple comparisons test). ^∗^*P* < 0.05; ^∗∗^*P* < 0.01; ^∗∗∗^*P* < 0.001; n.s., not significant. NA, not applicable. “!” Indicates rough estimates of EC_50_ from the available data in cases when maximum response was not reached. **(J)** The plot shows dissociation constants (*K*_d_) obtained from isothermal titration calorimetry, *n* = 2–5. ^∗∗∗^*P* < 0.001; ^****^P < 0.0001 (one-way ANOVA, Tukey’s multiple comparisons test).

Next, we tested the dependence of FGF2-STABs on heparin/HS for induction of FGF signaling using BaF3 cells that naturally do not express heparin/HS. First, we tested the ability of FGF2 variants to induce proliferation of BaF3-FGFR2c with and without the addition of heparin to the medium. To this end, BaF3-FGFR2c cells were seeded in basal medium containing serum and treated with FGF2 variants in the presence (2 μg/ml) or absence of heparin for 24 h (Figure 4A). When the cells were cultured in the medium with serum, all FGF2 variants were able to induce proliferation of BaF3-FGFR2c even without the addition of heparin to the medium. FGF2-wt showed a 3.7-fold increase in EC_50_, FGF2-STAB1 a 3.7-fold increase and FGF2-STAB2 a 41-fold increase in the absence of heparin (Figures 4B,C). However, the EC_50_ values of FGF2-STABs without heparin were still an order of magnitude lower than the EC_50_ of FGF2-wt with heparin.

Because serum can contain heparin/HS, we performed the BaF3-FGFR2c proliferation test in serum-free medium with and without the addition of heparin. BaF3-FGFR2c cell proliferation was measured after 2 days of incubation with FGF2 variants (Figure 4D). Under these conditions, the EC_50_ values significantly increased for all FGF2 variants (Figures 4E,F). FGF2-wt practically lost its ability to effectively induce signaling in the absence of heparin, while FGF2-STABs still retained some capacity to induce FGFR signaling, although it was weak. We also investigated the requirement of heparin for induction of FGF signaling by FGF2 variants using protamine sulfate, an inhibitor of heparin (Wolzt et al., 1995). BaF3-FGFR2c cells were seeded in serum-containing medium with heparin and incubated with FGF2 variants in the presence or absence of protamine sulfate for 2 days (Figure 4G). We observed a global 2-fold increase of EC_50_ for all FGF2 variants (Figures 4H,I). Taken together, these results revealed that FGF2-STABs are much less dependent on heparin for efficient induction of FGFR signaling.

Decreased dependence of FGF2-STABs on heparin for induction of FGFR signaling could potentially stem from their altered affinity to heparin. Therefore, we tested the affinity of FGF2 variants to heparin by measurement of *K*_d_ for the binding interactions between FGF2 variants and heparin using isothermal titration calorimetry. FGF2-wt exhibited the lowest *K*_d_ value (0.15 μM), while FGF2-STAB1 and FGF2-STAB2 exhibited 3.5- and 6.2-fold higher *K*_d_ than FGF2-wt, respectively (Figure 4J and Table 2). This indicates that FGF2-STABs have a significantly lower affinity to heparin. Because FGF2-STABs exhibited a fully preserved FGFR specificity and an even better efficiency in the induction of BaF3-FGFR cell proliferation, the reduced binding affinity of FGF2-STABs to heparin represents another indication of their lower dependence on heparin for FGFR signaling.

**TABLE 2:** Dissociation constants (*K*_d_) for binding of FGF2 variants to heparin.

**FGF2 variant**	***K*_d_ [μM]**
FGF2-wt	0.15 ± 0.02
FGF2-STAB1	0.53 ± 0.03
FGF2-STAB2	0.93 ± 0.10

*The values represent the mean ± SD (*n* = 2–5).*

### FGF2-STAB Is More Efficient Than FGF2-wt at Inducing ERK1/2 Signaling at Low Concentrations in BaF3-FGFR Cells

**FIGURE 5:**
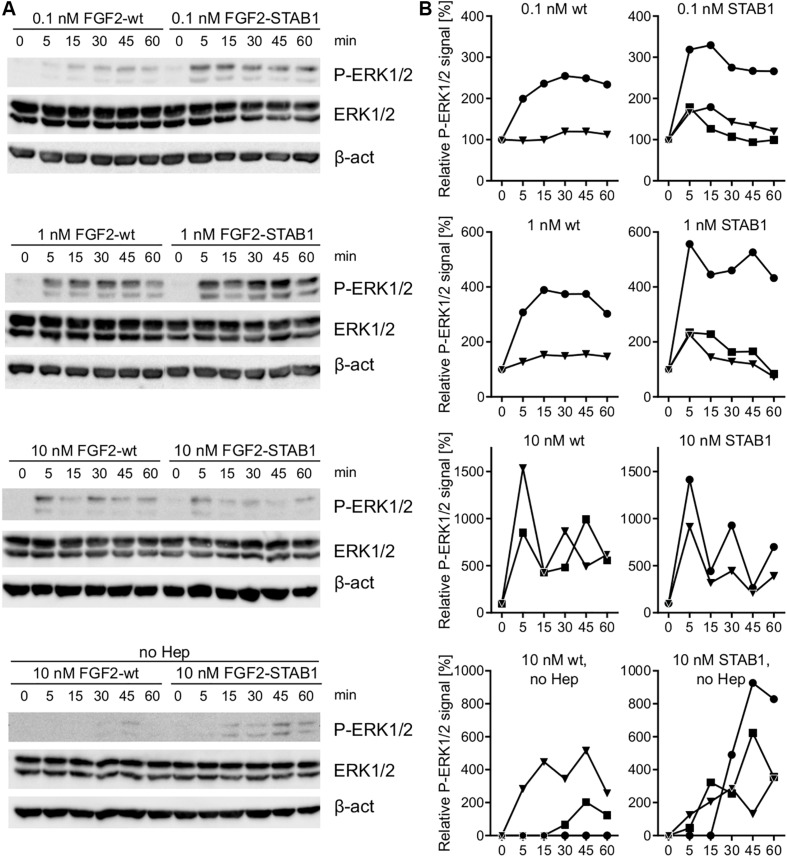
In BaF3-FGFR2c cells and in the presence of heparin, FGF2-STAB1 is more efficient at inducing ERK1/2 signaling at low concentrations than FGF2-wt. **(A,B)** Analysis of ERK1/2 phosphorylation dynamics in response to FGF2 variants in BaF3-FGFR2c cells by Western blot. **(A)** Representative photographs of Western blot analysis of ERK1/2 phosphorylation in response to 0.1–10 nM FGF2-wt, or FGF2-STAB1 in the presence of 2 μg/ml heparin (Hep), and to 10 nM FGF2 variants in the absence of heparin. P-ERK1/2 (Thr202/Tyr204), ERK, and β-actin (β-act) signals were detected on a single blot. **(B)** Graphical presentation of ERK1/2 phosphorylation dynamics. The line plots indicate the relative amount of ERK1/2 phosphorylation, normalized to total ERK1/2. Each line represents one experiment (an independent biological replicate).

Our FGFR-specificity and thermostability studies in the BaF3-FGFR cells showed that FGF2-STABs promote BaF3-FGFR cell proliferation at much lower concentrations than FGF2-wt. This suggested that at low concentrations, FGF2-STABs induced FGFR signaling more efficiently than FGF2-wt. To test this, we investigated ERK1/2 signaling dynamics in BaF3-FGFR cells because previous studies showed that ERK1/2 signaling dynamics is the key determinant of cellular response to FGFR signaling (Zhu et al., 2010). More specifically, we analyzed ERK1/2 signaling dynamics in BaF3-FGFR2c and BaF3-FGFR1c cells in response to a range of FGF2-wt and FGF2-STAB1 concentrations (0.1–10 nM) by Western blot detection of activated ERK1/2 (phosphorylated on Thr202/Tyr204; P-ERK1/2) at time points up to 60 min after addition of FGF2. We found out that in BaF3-FGFR2c cells treated with 0.1 or 1 nM FGF2 in the presence of heparin, FGF2-STAB1 induced ERK1/2 signaling with a higher amplitude and with faster dynamics than FGF2-wt (Figures 5A,B). Similarly, in BaF3-FGFR1c cells, FGF2-STAB1 induced ERK1/2 signaling with a higher amplitude than FGF2-wt at both 0.1 and 1 nM FGF2 in the presence of heparin, and the dynamics of full ERK1/2 activation was faster in response to FGF2-STAB than FGF2-wt at 0.1 nM concentration (Supplementary Figures 3A,B).

In response to 10 nM FGF2 in the presence of heparin, the ERK1/2 signaling amplitude and dynamics was similar between FGF2-wt and FGF2-STAB1 in BaF3-FGFR2c cells and, interestingly, we detected fluctuations in P-ERK1/2 level, rather than gradual decrease (Figures 5A,B). Such fluctuations were not observed in BaF3-FGFR1c cells, yet 10 nM FGF2-wt induced a higher amplitude of ERK1/2 signaling than FGF2-STAB1 and the ERK signaling dynamics showed irregularities, with rather abrupt downregulation of ERK1/2 activity within 15–30 min after stimulation, followed by a gradual increase in ERK1/2 activity (Supplementary Figures 3A,B). The fluctuations and irregularities in ERK1/2 signaling dynamics could indicate that 10 nM concentration represents a concentration close to a maximal concentration, at which the cells are able to elicit sustained ERK1/2 signaling that is required for proliferative response. Correspondingly, at 10 nM FGF2, we observed a plateau in cell proliferative response in both BaF3-FGFR1c and BaF3-FGFR2c cells (Figure 1B).

In the absence of heparin, both 10 nM FGF2-wt and 10 nM FGF2-STAB1 were able to induce ERK1/2 signaling in BaF3-FGFR2c cells, although for both FGF2-wt and FGF2-STAB1, the signaling amplitude was lower and the dynamics of reaching signaling maximum was slower in the absence than in the presence of heparin (Figures 5A,B). Moreover, in the absence of heparin, FGF2-STAB1 exhibited a faster dynamics and higher amplitude of ERK1/2 signaling activation than FGF2-wt (Figure 5A). These differences in ERK1/2 signaling dynamics correlated with the cellular proliferative response to FGF2-wt and FGF2-STABs in the presence and absence of heparin (Figure 4B). Interestingly, in BaF3-FGFR1c cells, neither FGF2-wt nor FGF2-STAB1 induced ERK1/2 signaling in the absence of heparin (Supplementary Figures 3A,B).

### FGF2-STABs Are More Efficient Than FGF2-wt at Inducing ERK1/2 Signaling at Low Concentrations in BaF3-FGFR Cells

Because BaF3-FGFR cells represent an engineered system of FGFR expression, next we tested the efficacy of FGF2 variants in cells that naturally express FGFRs, primary mammary fibroblasts. Previous studies have shown that in cells naturally expressing FGFRs, FGF ligands elicit a biphasic mitogenic response, stimulating cell proliferation at optimal concentration, but failing to do so at high concentration (Fox et al., 1988; Ke et al., 1990; Zhu et al., 2010), and that ERK1/2 signaling dynamics is the key determinant of cellular response (Zhu et al., 2010). Therefore, we investigated ERK1/2 signaling dynamics in response to a wide range of FGF2-wt, FGF2-STAB1 and FGF2-STAB2 concentrations by Western blot detection of activated ERK1/2 (phosphorylated on Thr202/Tyr204; P-ERK1/2) at time points up to 60 min after addition of FGF2.

**FIGURE 6:**
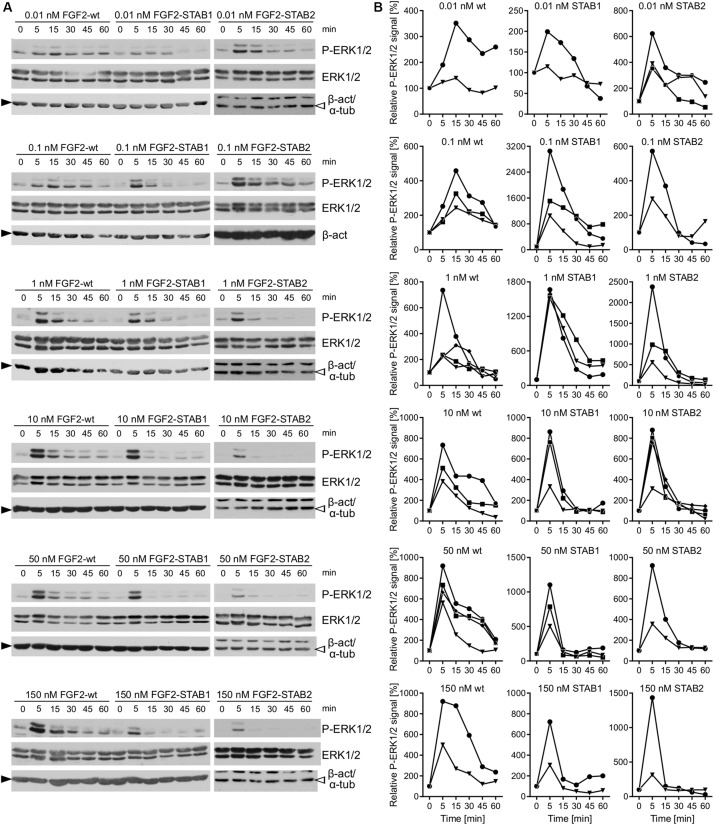
FGF2-STABs are more efficient at inducing ERK1/2 signaling at low concentrations. **(A,B)** Analysis of ERK1/2 phosphorylation dynamics in response to FGF2 variants in primary fibroblasts by Western blot. **(A)** Representative Western blots of ERK1/2 phosphorylation in response to 0.01–150 nM FGF2-wt, FGF2-STAB1, or FGF2-STAB2. P-ERK1/2 (Thr202/Tyr204), ERK, and β-actin (β-act; full black arrowhead) or α-tubulin (α-tub; empty arrowhead) signals were detected on a single blot. **(B)** Graphical presentation of ERK1/2 phosphorylation dynamics in response to FGF2-wt, FGF2-STAB1 and FGF2-STAB2. The line plots indicate the relative amount of ERK1/2 phosphorylation, normalized to total ERK1/2. Each line represents one experiment (an independent biological replicate).

When the fibroblasts were treated with FGF2 variants with no addition of heparin to the medium, thus the FGF2 signaling was dependent only on HS naturally expressed by the fibroblasts, we detected activation of ERK1/2 signaling in response to as low as 0.01 nM and as high as 150 nM FGF2 concentration for all FGF2 variants (Figures 6A,B). Importantly, we registered major differences in ERK1/2 signaling dynamics between FGF2-wt and the FGF2-STABs. In response to FGF2-wt, ERK1/2 signaling activation reached a maximum at 5–15 min after FGF2 treatment and then it gradually decreased (Figures 6A,B). At 0.01 or 0.1 nM FGF2-wt, ERK1/2 phosphorylation peaked at 15 min. At 1 nM FGF2-wt or higher, ERK1/2 phosphorylation reached maximum sooner, at 5 min after FGF2 treatment (Figures 6A,B).

In response the FGF2-STAB1 or FGF2-STAB2, ERK1/2 signaling peaked at 5 min with as little as 0.01 nM FGF2-STAB1 or FGF2-STAB2 (Figures 6A,B). Also, the amplitude of ERK1/2 signaling in response to FGF2-STABs was at low concentrations (0.01–1 nM) higher than the amplitude of ERK1/2 signaling in response to FGF2-wt at the respective concentration (Figures 6A,B). These data revealed that at concentrations to 1 nM, FGF2-STABs induced ERK1/2 signaling more potently, with a faster dynamics of full ERK1/2 activation and higher amplitudes of ERK1/2 signaling than FGF2-wt. At concentrations 10 nM and higher, the amplitudes of ERK1/2 signaling were similar for FGF2-wt and FGF2-STABs. Interestingly, at 10 nM or higher concentrations, FGF2-STABs elicited only a transient peak of ERK1/2 activation (at 5 min), followed by a sharp P-ERK1/2 signal decay to the baseline level (corresponding to unstimulated cells) or even below it (Figures 6A,B). Therefore, 10 nM and higher concentrations represent supramaximal concentrations of FGF2-STABs that are unable to elicit sustained ERK1/2 signaling.

### In the Absence of External Heparin, FGF2-wt Induces a Sigmoidal Response and FGF2-STABs Induce a Biphasic Response in Primary Fibroblasts

**FIGURE 7:**
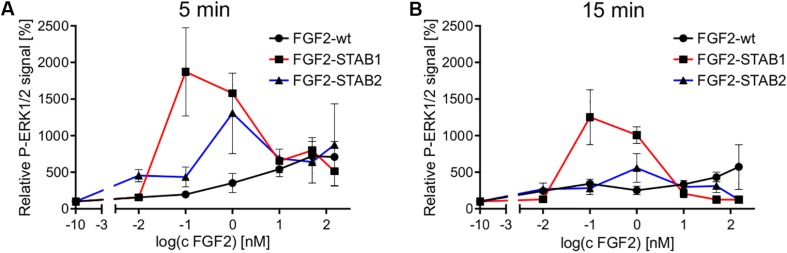
Dose-response profile of ERK1/2 activation displays sigmoidal response to FGF2-wt and biphasic response to FGF2-STABs in the absence of heparin. Comparison of ERK1/2 phosphorylation at 5 min **(A)** or 15 min **(B)** after FGF2 treatment in primary fibroblasts. The line plots represent mean ± SEM of data represented in Figure 6 (*n* = 2–4).

Analysis of the dose-response curves revealed that FGF2-STABs induced a biphasic response, while FGF2-wt induced a sigmoidal response in the primary fibroblasts (Figure 7). This observation correlated with the detection of supramaximal concentrations for FGF2-STABs, while no such concentration-dependent signaling limits were detected for FGF2-wt (Figures 6A,B). FGF2-STABs were more efficient than FGF2-wt at inducing ERK signaling at low concentrations and displayed EC_50_ values approximately 10–60 times lower than FGF2-wt in primary fibroblasts, as revealed from the analysis of the sigmoid parts of the dose-response curves (Supplementary Figure 4).

When heparin was used during the FGF2 treatment, most of the FGF2 concentration-dependent differences in ERK1/2 signaling dynamics between FGF2-wt and FGF2-STABs diminished (Supplementary Figures 5A,B). FGF2-wt was able to elicit the maximum ERK1/2 activation at 5 min after FGF2 treatment at as little as 0.001 nM concentration, similar to FGF2-STABs. Importantly, in the presence of heparin, FGF2-STABs showed similar pattern of ERK1/2 dynamics as FGF2-wt at the whole range of FGF2 concentrations tested (0.001–50 nM). After reaching the maximum ERK1/2 signaling activation at 5 min after FGF2 treatment, the ERK1/2 phosphorylation decreased gradually for all FGF2-wt, FGF2-STAB1 and FGF2-STAB2. No abrupt P-ERK1/2 signal decay was observed, such as that observed for the FGF2-STABs at supramaximal concentrations in the absence of heparin. In fact, in the presence of heparin, all FGF2 variants induced sustained ERK1/2 signaling that was more reluctant in returning to the baseline level. Analysis of the dose-response curves revealed that in the presence of heparin, all FGF2 variants induced a sigmoidal response in primary fibroblasts (Supplementary Figure 6). Moreover, in the presence of heparin, the amplitude of ERK1/2 signaling was more similar between FGF2-wt and FGF2-STABs than without heparin (Supplementary Figure 7). Our data indicate that exogenous heparin is a major modulator of FGFR responsiveness to FGF2 variants and ERK1/2 signaling dynamics.

## Discussion

In this study, we first provided a detailed characterization of the FGF2-STABs’ receptor specificities, thermal stability, and heparin requirement for their biological activity, and then used the FGF2-STABs to investigate the effect of FGF2 stability on ERK1/2 signaling dynamics. Our tests of the FGFR specificity in the BaF3 cells showed that the FGFR specificity of the FGF2-STABs was not compromised by the introduction of the stabilizing mutations. Both FGF2-STAB1 and FGF2-STAB2 showed the same FGFR specificity as FGF2-wt. Moreover, the tests of FGFR specificity revealed a significantly decreased EC_50_ of FGF2-STABs in comparison to FGF2-wt.

Using the proliferative response of BaF3-FGFR cells, we also analyzed thermal stability of the FGF2 variants. We observed that FGF2-STABs were significantly more stable than FGF2-wt after various thermal treatments, confirming and elaborating the first report on FGF2-STABs (Dvorak et al., 2018). Moreover, we found that the inherent stability of FGF2-STABs resembled the stability of FGF2-wt in the presence of heparin. In fact, the inherent stability of FGF2-STABs was so high that heparin had only a minor stabilization effect on FGF2-STABs during pre-incubations. Similar conclusions came from differential scanning fluorimetry.

The decreased dependence of FGF2-STABs on heparin for stabilization correlated with their decreased affinity to heparin and decreased dependence on heparin for induction of FGFR and ERK1/2 signaling. Our observations are in agreement with a previous study that showed that FGF1 variants with increased stability were less dependent on heparin binding for induction of FGF-FGFR complex formation and FGFR signaling (Zakrzewska et al., 2009). Several studies suggested that the main role of heparin/HS in FGFR signaling is to protect FGF against proteolytic degradation (Gospodarowicz and Cheng, 1986; Zakrzewska et al., 2009) and to stabilize the FGF2-FGFR complex (Fannon and Nugent, 1996; Lundin et al., 2003), and that the FGF2-FGFR complex can form in the absence of heparin/HS (Roghani et al., 1994; Fannon and Nugent, 1996).

Analysis of the ERK1/2 signaling in BaF3-FGFR2c and BaF3-FGFR1c cells revealed that at low concentrations (0.1–1 nM FGF2) and in the presence of heparin, FGF2-STAB1 induced ERK1/2 signaling with a higher amplitude and/or a faster dynamics of reaching the signaling maximum than FGF2-wt. Therefore, FGF2-STAB1 was more efficient at inducing ERK1/2 signaling than FGF2-wt. At 10 nM concentration in the presence of heparin, the differences in ERK1/2 signaling amplitude and dynamics between FGF2-STAB1 and FGF2-wt diminished. Moreover, at 10 nM concentration, the ERK1/2 signaling dynamics exhibited fluctuations and irregularities, probably due to difficulties in proper formation of FGF2-heparin-FGFR complexes and/or assembly of downstream signaling modules (Zhu et al., 2010), which indicated that 10 nM might be the maximum borderline concentration for both wild-type and hyperstable FGF2 variants to elicit a cellular response in the BaF3-FGFR cells. Importantly, the efficiency of ERK1/2 signaling induction by FGF2 variants, revealed by the patterns of ERK1/2 signaling dynamics and amplitude, corresponded to the BaF3-FGFR1c and BaF3-FGFR2c cellular proliferative response.

In primary mammary fibroblasts, which express FGFR1c and FGFR2c isoforms (Sumbal and Koledova, 2019), FGF2-wt and FGF2-STABs exhibited differences in dynamics and amplitude of ERK1/2 signaling activation when no heparin was added and the cells were dependent only on their own HS for FGFR signaling activation. Under these conditions, FGF2-STABs were more potent activators of ERK1/2 signaling, capable of inducing faster and/or stronger ERK1/2 activation at low concentrations (0.01–1 nM) than FGF2-wt. Importantly, at these concentrations, the inactivation dynamics of ERK1/2 phosphorylation were similar between FGF2-wt and FGF2-STABs, with no signs of abnormally slow signal decay, which would indicate cancer-like dynamics (Bugaj et al., 2018).

Moreover, at 10 nM concentration, the FGF2-STABs reached the upper limit of their ability to induce sustained ERK1/2 phosphorylation (supramaximal concentration) and induced only a transient spike of ERK1/2 phosphorylation at 5 min after FGF2 treatment that quickly dropped within the next 10 min. In contrast, FGF2-wt efficiently induced a sustained ERK1/2 phosphorylation (with the peak of ERK1/2 phosphorylation at 5 min and gradual decrease over 60 min after FGF2 treatment) even at 150 nM concentration. Supramaximal concentration values appear to be not only protein-type but also cell-type dependent. Previous studies reported supramaximal concentrations of FGF2 to be 1.25 nM in human lung epithelial cancer cells (H1703) (Kanodia et al., 2014), 5.6 nM in rat mammary fibroblast cells (Rama 27) (Zhu et al., 2010), 31.25 nM in human metastatic breast cancer cell line (MDA-MB-134) (Kanodia et al., 2014), and 56 nM in primary human umbilical vein endothelial cells (HUVEC) (Fox et al., 1988). The cell-type dependent differences in supramaximal concentrations (as well as EC_50_ values) can be caused by cell-specific differences in FGFR and HS proteoglycan expression, HS biosynthesis and HS modification that affect FGF2 binding and biological activities (Turnbull et al., 1992; Ishihara et al., 1994; Bishop et al., 2007; Lai et al., 2008; Kanodia et al., 2014). Moreover, our results point out the striking differences in FGF signaling between the primary cells used in this study (primary mouse mammary fibroblasts) and immortalized cell lines used in other studies, and they demonstrate the importance of result validation and hypothesis testing using physiologically more relevant *in vitro* models.

The differences between FGF2-wt and FGF2-STABs in ERK1/2 signaling dynamics in the short-time experiments were unlikely to be due to the increased stability of FGF2-STABs *per se*, i.e., decreased ligand degradation in comparison to FGF2-wt. Instead, the differences in ERK1/2 signaling dynamics stemmed, most likely, from the increased affinity of FGF2-STABs to FGFR and the decreased dependence on heparin/HS for FGF2-FGFR complex formation. This could lead to an altered dynamics of FGF2-HS-FGFR complex formation and stabilization, differences in efficiency of fibroblast growth factor receptor substrate 2 (FRS2) phosphorylation, and subsequent recruitment of growth factor receptor-bound protein 2 via FRS2 to FGFR that is required for sustained ERK1/2 phosphorylation (Zhu et al., 2010). We propose two possible causes of alterations in FGF2-FGFR complex formation by FGF2-STABs: (i) increased efficiency of FGF2-STABs to form FGF2-FGFR complexes and (ii) decreased dependence of FGF2-STABs on heparin/HS for FGF2-FGFR complex stabilization. Both mechanisms have support in the study that showed that FGF1 mutants with increased stability were more efficient in the induction of FGF-FGFR complex formation and FGFR signaling (Zakrzewska et al., 2009). It would be interesting to further test the role of FGF2 protein stability and affinity to FGFR using a computational model for FGF signaling (Kanodia et al., 2014).

Addition of heparin during the FGF2 treatment of primary fibroblasts diminished the differences in ERK1/2 signaling dynamics between FGF2-wt and FGF2-STABs. Heparin stabilized FGF2-wt and enabled a fast and efficient ERK1/2 signaling activation also at low concentrations (0.01–1 nM). This demonstrated that heparin is a major modulator of FGFR responsiveness to FGF2 variants and ERK1/2 signaling dynamics.

FGF2-STABs provide the advantage of about 10- to 100-times lower EC_50_ values and prolonged availability to the cells due to an increased thermal stability in comparison to FGF2-wt. These characteristics make FGF2-STABs a valuable material for applications, where high and/or sustained FGF2 concentrations are required, such as human embryonic stem cell (hESC) culture (Lotz et al., 2013) or therapeutic applications (Matsumoto et al., 2013). The previous report on hyperstable FGF2 variants demonstrated increased proficiency of FGF2-STABs to promote hESC proliferation (Dvorak et al., 2018). Currently several preclinical studies are investigating the use of FGF2-STABs in wound healing applications. Importantly, the high stability of FGF2-STABs that is largely independent on heparin makes FGF2-STABs a valuable tool in therapeutic applications where use of heparin is contraindicated or not desired. We also propose the potential use of FGF2-STABs in tissue culture and engineering. Because the intensity, duration and gradients of FGF2 signaling are important determinants of developmental outcomes *in vivo* and cell behavior *in vitro* (Serls et al., 2005; Roszell et al., 2009; Ameri et al., 2010; Wu et al., 2014), any potential use of FGF2-STABs in established protocols, such as for stem cell culture, directed differentiation of cells, organoid formation, or in tissue engineering requires additional testing to determine optimal FGF2-STAB concentration and treatment duration.

## Data Availability Statement

All datasets generated for this study are included in the article/Supplementary Material.

## Ethics Statement

The animal study was reviewed and approved by the Ministry of Agriculture of the Czech Republic, and the Expert Committee for Laboratory Animal Welfare at the Faculty of Medicine, Masaryk University.

## Author Contributions

ZK: conception and design of the study. ZK, JS, AR, RC, and GL: acquisition, analysis, and interpretation of the data. BH and JD: provision of materials. ZK, VS, and JD: funding acquisition. ZK: drafting of the manuscript. ZK, JS, RC, BH, and JD: critical revision of the manuscript. All authors: approval of the final version.

## Conflict of Interest

RC, JD, and VS are the shareholders of Enantis Ltd. GL and BH are the employees of Enantis Ltd.

The remaining authors declare that the research was conducted in the absence of any commercial or financial relationships that could be construed as a potential conflict of interest.

## Abbreviations

EC_50_: half-maximal effective concentration
ERK: extracellular-signal − regulated kinase
FGF2: fibroblast growth factor 2
FGF2-STAB: stabilized FGF2
FGF2-wt: wild-type FGF2
FGFR: fibroblast growth factor receptor
FRS2: fibroblast growth factor receptor substrate 2
hESC: human embryonic stem cells
HS: heparan sulfate
*K*_d_: dissociation constant
SC_50_: half-maximal stabilization concentration
*T*_m_: melting temperature.
