# Supplementary material for: Fibroblast Growth Factor 2 Protein Stability Provides Decreased Dependence on Heparin for Induction of FGFR Signaling and Alters ERK Signaling Dynamics
Source: Front Cell Dev Biol. 2019 Dec 12;7:331. doi: 10.3389/fcell.2019.00331 (PMC6924264; doi:10.3389/fcell.2019.00331)
Supplement: Supplementary file 1 [file Data_Sheet_1.pdf]

Supplementary Material

1 Supplementary Figures and Tables

1.1 Supplementary Figures

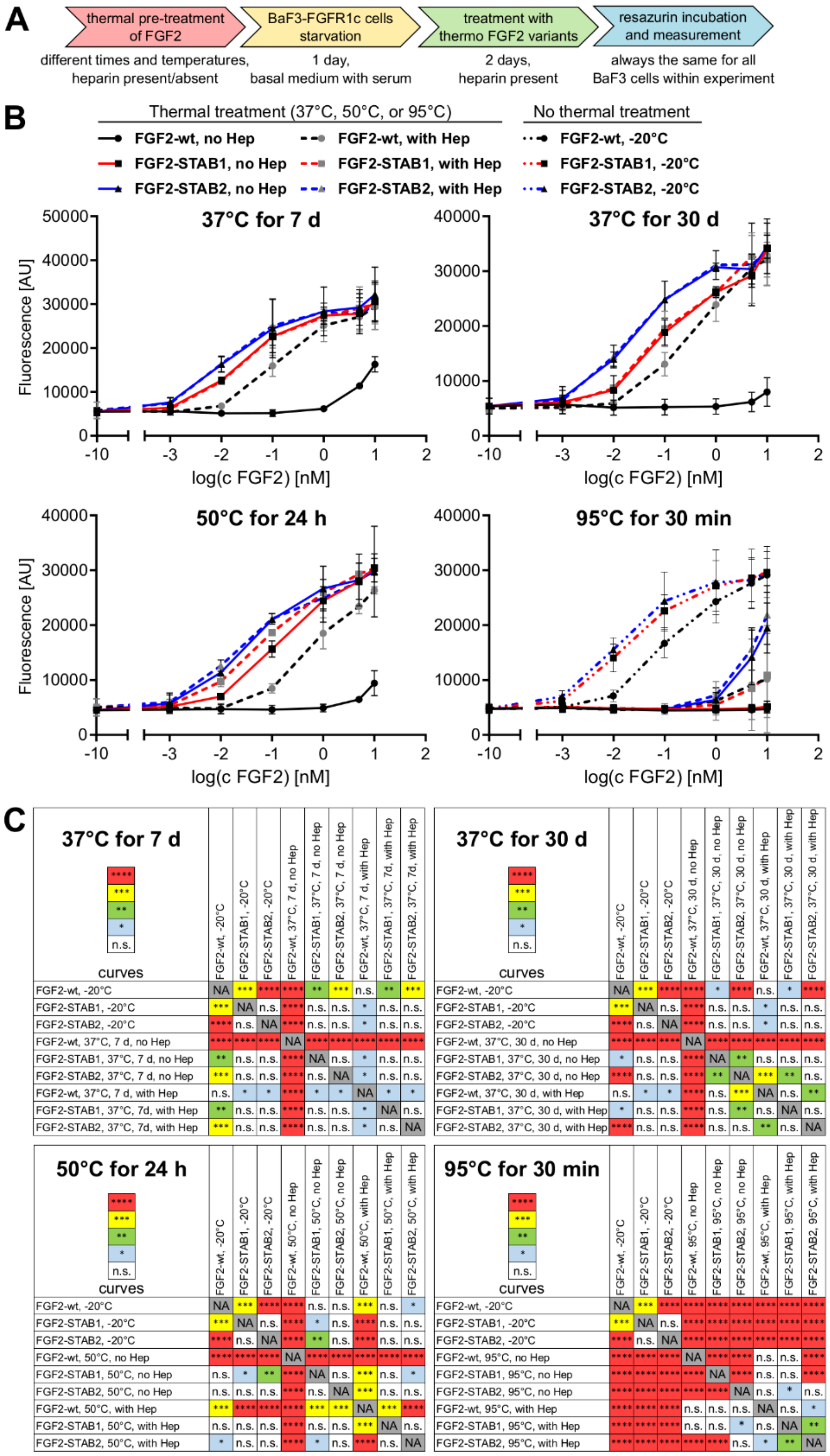

SUPPLEMENTARY FIGURE 1 | FGF2-STABs show increased thermal stability. **(A-C)** Thermostability testing using proliferation assay on BaF3-FGFR1c cells. **(A)** Experimental design scheme. FGF2 variants were exposed to 37°C, 50°C, or 95°C in the presence (2 µg/ml heparin; with Hep) or absence of heparin (no Hep) for the indicated time, or not thermally treated at all but stored at -20°C, and then used to treat the BaF3-FGFR1c cells. BaF3-FGFR1c cells were seeded in basal medium containing serum and treated with FGF2 variants in the presence of heparin (2 µg/ml) for 4 days. **(B)** The line plots show resorufin fluorescence, measured after 4 days of culture with FGF2 variants, as mean ± SD, *n* = 2-3. For visual clarity, the plots for thermally non-treated FGF2 variants (-20°C) are shown only in the plots with 95°C-treated variants, otherwise they were too much overlapping with the curves in graphs of other thermal treatments. **(C)** The color

maps show results of statistical comparison of curves from A. *$P < 0.05$; **$P < 0.01$; ***$P < 0.001$; ****$P < 0.0001$; n.s., not significant (two-way ANOVA). NA, not applicable.

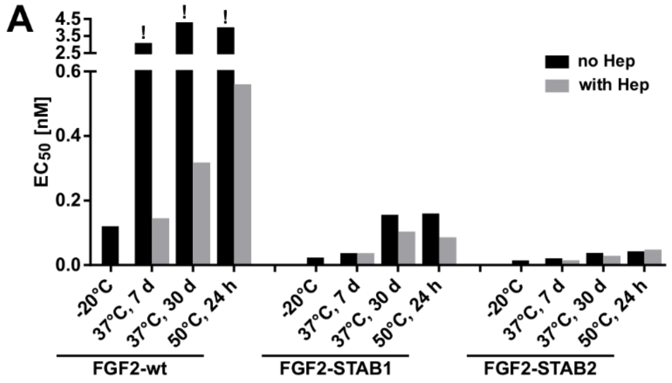

B

37°C for 7 d (EC50; legend: **** / *** / ** / * / n.s.)

	FGF2-wt, -20°C	FGF2-STAB1, -20°C	FGF2-STAB2, -20°C	FGF2-wt, 37°C, 7 d, no Hep	FGF2-STAB1, 37°C, 7 d, no Hep	FGF2-STAB2, 37°C, 7 d, no Hep	FGF2-wt, 37°C, 7 d, with Hep	FGF2-STAB1, 37°C, 7d, with Hep	FGF2-STAB2, 37°C, 7d, with Hep
FGF2-wt, -20°C	NA	***	***	****	*	**	n.s.	*	**
FGF2-STAB1, -20°C	***	NA	n.s.	****	n.s.	n.s.	**	n.s.	n.s.
FGF2-STAB2, -20°C	***	n.s.	NA	****	n.s.	n.s.	n.s.	n.s.	n.s.
FGF2-wt, 37°C, 7 d, no Hep	****	****	****	NA	****	****	****	****	****
FGF2-STAB1, 37°C, 7 d, no Hep	*	n.s.	n.s.	****	NA	n.s.	**	n.s.	n.s.
FGF2-STAB2, 37°C, 7 d, no Hep	**	n.s.	n.s.	****	n.s.	NA	**	n.s.	n.s.
FGF2-wt, 37°C, 7 d, with Hep	n.s.	**	n.s.	****	**	**	NA	*	**
FGF2-STAB1, 37°C, 7d, with Hep	*	n.s.	n.s.	****	n.s.	n.s.	*	NA	n.s.
FGF2-STAB2, 37°C, 7d, with Hep	**	n.s.	n.s.	****	n.s.	n.s.	**	n.s.	NA

37°C for 30 d (EC50; legend: **** / *** / ** / * / n.s.)

	FGF2-wt, -20°C	FGF2-STAB1, -20°C	FGF2-STAB2, -20°C	FGF2-wt, 37°C, 30 d, no Hep	FGF2-STAB1, 37°C, 30 d, no Hep	FGF2-STAB2, 37°C, 30 d, no Hep	FGF2-wt, 37°C, 30 d, with Hep	FGF2-STAB1, 37°C, 30 d, with Hep	FGF2-STAB2, 37°C, 30 d, with Hep
FGF2-wt, -20°C	NA	***	***	****	n.s.	*	****	n.s.	*
FGF2-STAB1, -20°C	***	NA	n.s.	****	*	n.s.	****	n.s.	n.s.
FGF2-STAB2, -20°C	***	n.s.	NA	****	*	n.s.	****	*	n.s.
FGF2-wt, 37°C, 30 d, no Hep	****	****	****	NA	****	****	****	****	****
FGF2-STAB1, 37°C, 30 d, no Hep	n.s.	*	*	****	NA	n.s.	***	n.s.	n.s.
FGF2-STAB2, 37°C, 30 d, no Hep	*	n.s.	n.s.	****	n.s.	NA	***	n.s.	n.s.
FGF2-wt, 37°C, 30 d, with Hep	****	****	****	****	***	***	NA	****	****
FGF2-STAB1, 37°C, 30 d, with Hep	n.s.	n.s.	*	****	n.s.	n.s.	****	NA	n.s.
FGF2-STAB2, 37°C, 30 d, with Hep	*	n.s.	n.s.	****	n.s.	n.s.	****	n.s.	NA

50°C for 24 h (EC50; legend: **** / *** / ** / * / n.s.)

	FGF2-wt, -20°C	FGF2-STAB1, -20°C	FGF2-STAB2, -20°C	FGF2-wt, 50°C, 24 h, no Hep	FGF2-STAB1, 50°C, 24 h, no Hep	FGF2-STAB2, 50°C, 24 h, no Hep	FGF2-wt, 50°C, 24 h, with Hep	FGF2-STAB1, 50°C, 24 h, with Hep	FGF2-STAB2, 50°C, 24 h, with Hep
FGF2-wt, -20°C	NA	**	***	****	n.s.	n.s.	***	n.s.	n.s.
FGF2-STAB1, -20°C	**	NA	n.s.	****	**	n.s.	****	n.s.	n.s.
FGF2-STAB2, -20°C	***	n.s.	NA	****	***	n.s.	****	n.s.	n.s.
FGF2-wt, 50°C, 24 h, no Hep	****	****	****	NA	****	****	***	***	***
FGF2-STAB1, 50°C, 24 h, no Hep	n.s.	**	***	****	NA	n.s.	n.s.	n.s.	n.s.
FGF2-STAB2, 50°C, 24 h, no Hep	n.s.	n.s.	n.s.	****	n.s.	NA	n.s.	n.s.	n.s.
FGF2-wt, 50°C, 24 h, with Hep	***	****	****	***	n.s.	n.s.	NA	**	***
FGF2-STAB1, 50°C, 24 h, with Hep	n.s.	n.s.	n.s.	***	n.s.	n.s.	**	NA	n.s.
FGF2-STAB2, 50°C, 24 h, with Hep	n.s.	n.s.	n.s.	***	n.s.	n.s.	***	n.s.	NA

SUPPLEMENTARY FIGURE 2 | FGF2-STABs show increased thermal stability, as indicated by their lower EC_{50} values. **(A)** The bar plot presents EC_{50} values of FGF2 variants after thermal treatments, calculated from data presented in **Supplementary Figure 1A**. The exclamation mark (!) indicates EC_{50} values that are only rough estimates from the available data; however, true EC_{50} values are most likely higher because at the range of concentrations tested, maximum response was not reached. **(B)** The color maps show results of statistical analysis of EC_{50} values of FGF2 variants after thermal treatment calculated from data presented in **Supplementary Figure 1A**. *P < 0.05; **P < 0.01; ***P < 0.001; ****P < 0.0001; n.s., not significant (one-way ANOVA, Tukey's multiple comparisons test). NA, not applicable.

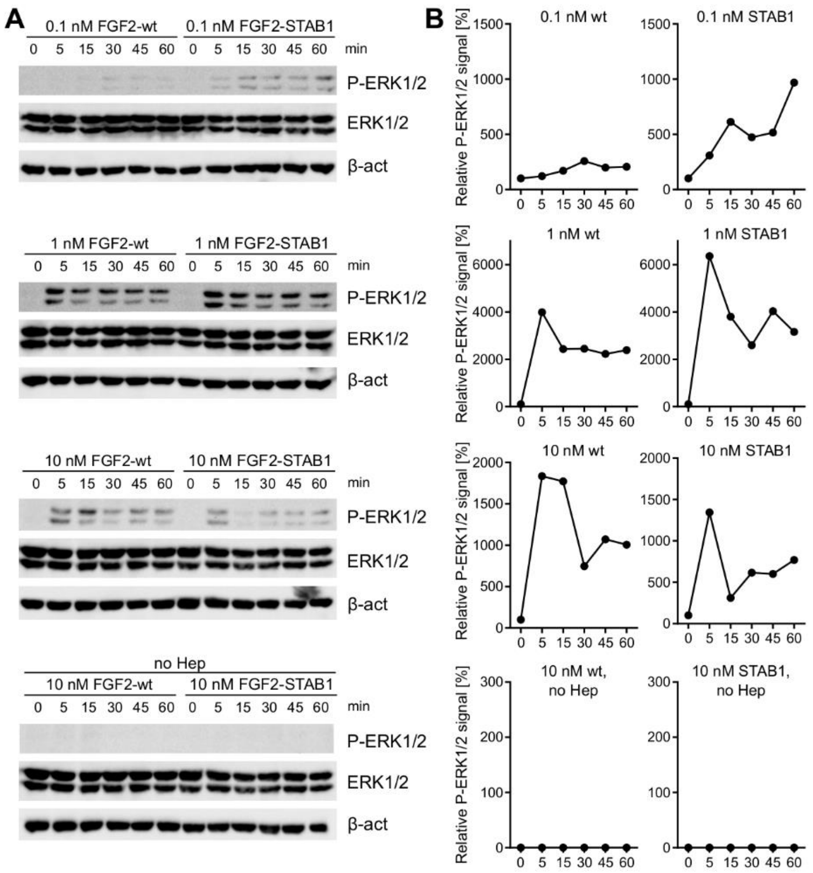

SUPPLEMENTARY FIGURE 3 | In BaF3-FGFR1c cells and in the presence of heparin, FGF2-STAB is more efficient at inducing ERK1/2 signaling at low concentrations than FGF2-wt. **(A, B)** Analysis of ERK1/2 phosphorylation dynamics in response to FGF2 variants in BaF3-FGFR1c cells by Western blot. **(A)** Representative photographs of Western blot analysis of ERK1/2 phosphorylation in response to 0.1 to 10 nM FGF2-wt, or FGF2-STAB1 in the presence of 2 µg/ml heparin (Hep), and to 10 nM FGF2 variants in the absence of heparin. P-ERK1/2 (Thr202/Tyr204), ERK, and β-actin (β-act) signals were detected on a single blot. **(B)** Graphical presentation of ERK1/2 phosphorylation dynamics. The line plots indicate the relative amount of ERK1/2 phosphorylation, normalized to total ERK1/2. Each line represents one experiment (an independent biological replicate).

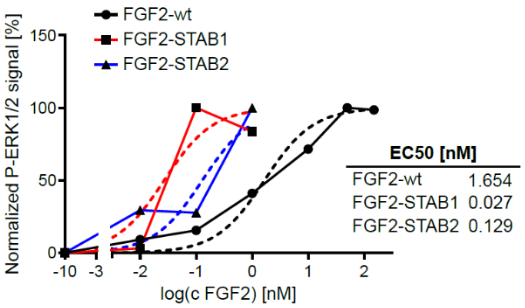

SUPPLEMENTARY FIGURE 4 | Calculation of the EC_{50} values of FGF2-wt, FGF2-STAB1 and FGF2-STAB2 in primary mammary fibroblasts. The EC_{50} values were calculated from the sigmoidal part of normalized dose-response curves of ERK1/2 phosphorylation at 5 min after addition of FGF2 in the absence of heparin. The plot is related to the data depicted in **Figures 5** and **6**.

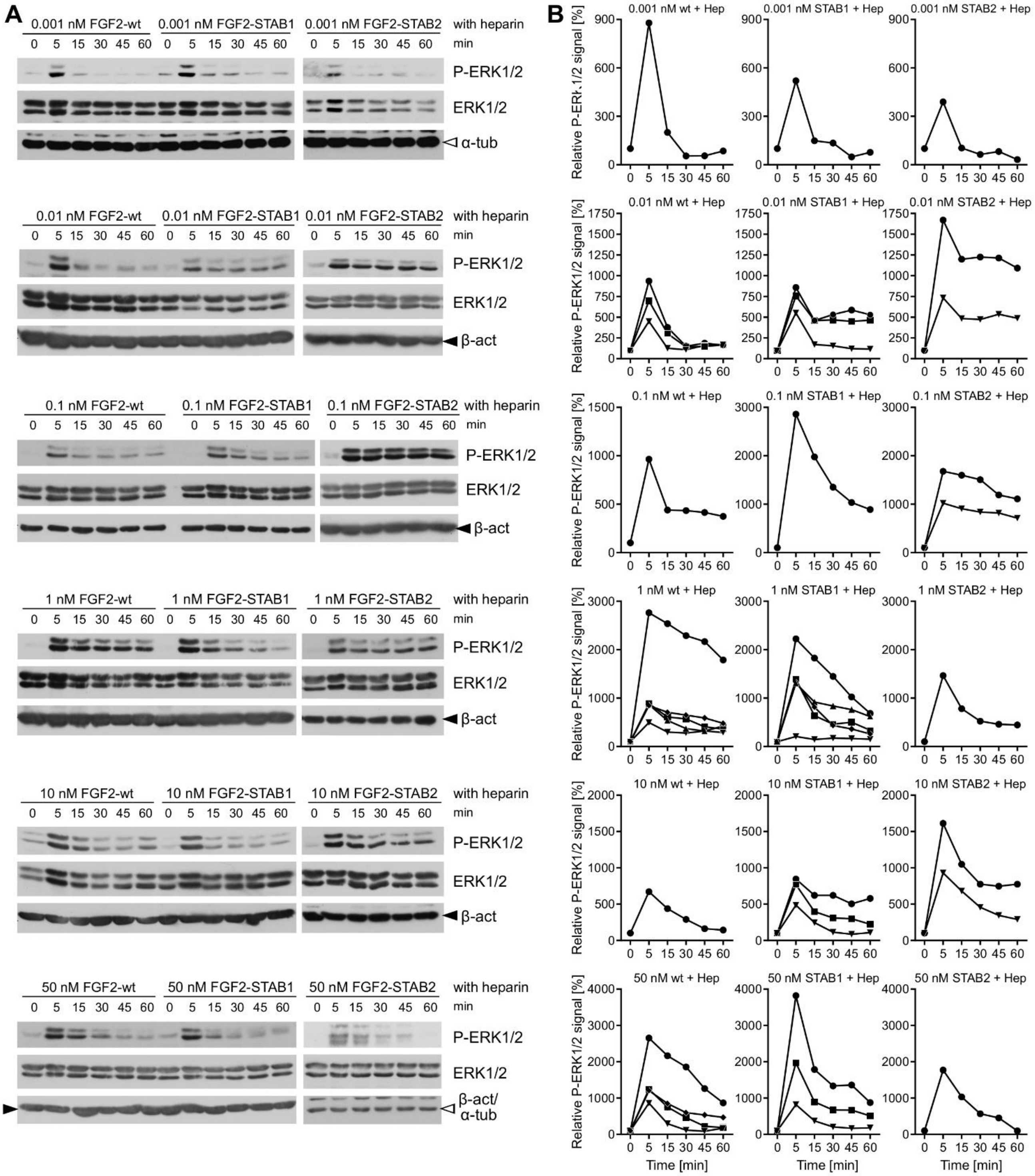

SUPPLEMENTARY FIGURE 5 | In the presence of heparin, FGF2-STABs and FGF2-wt induce similar ERK1/2 signaling response. **(A-C)** Analysis of ERK1/2 phosphorylation dynamics in response to FGF2 variants in the presence of heparin in primary fibroblasts by Western blot. **(A)** Representative photographs of Western blot analysis of ERK1/2 phosphorylation in response to 0.001 to 50 nM FGF2-wt, FGF2-STAB1, or FGF2-STAB2 and 4 μg/ml heparin. P-ERK1/2 (Thr202/Tyr204), ERK, and β-actin (β-act; full black arrowhead) or α-tubulin (α-tub; empty arrowhead) signals were detected on a single blot. **(B)** Graphical presentation of ERK1/2 phosphorylation dynamics. The line plots indicate the relative amount of ERK1/2 phosphorylation, normalized to total ERK1/2. Each line represents one experiment (an independent biological replicate).

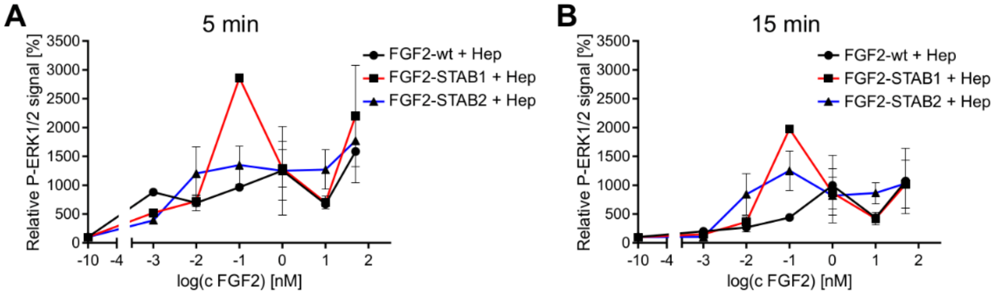

SUPPLEMENTARY FIGURE 6 | Dose-response profile of ERK1/2 activation after treatment with FGF2 variants and heparin. **(A, B)** Comparison of ERK1/2 phosphorylation at 5 min **(A)** or 15 min **(B)** after treatment with FGF2 and heparin (4 µg/ml) in primary fibroblasts. The line plots represent mean ± SEM of data represented in **Supplementary Figure 5** (*n* = 1-5).

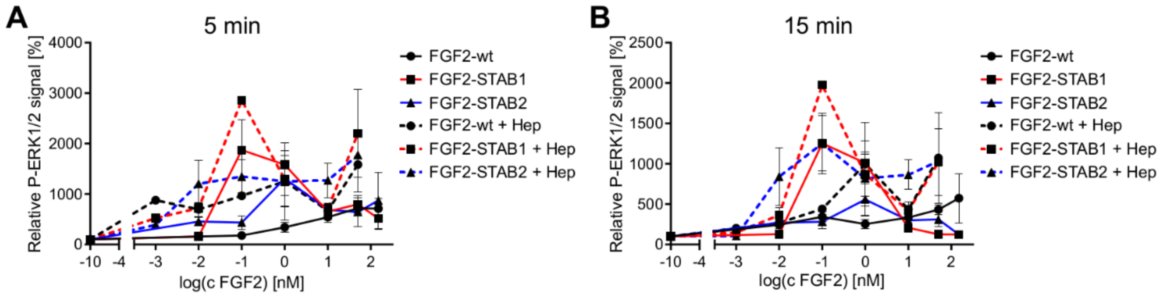

SUPPLEMENTARY FIGURE 7 | Dose-response profile of ERK1/2 activation after treatment with FGF2 variants, in the presence and absence of heparin. **(A, B)** Comparison of ERK1/2 phosphorylation at 5 min **(A)** or 15 min **(B)** after FGF2 treatment. The line plots represent mean ± SEM of data represented in **Figure 6** and **Supplementary Figure 5** (n = 1-5).

1.2 Supplementary Tables

SUPPLEMENTARY TABLE 1 | The dependence of EC_{50} values of FGF2 variants on thermal treatment and heparin. The EC_{50} values were calculated from experiments in BaF3-FGFR1c cells from plots depicted in **Supplementary Figure 1A**. The exclamation mark (!) indicates rough estimate values from the available data; however, true EC_{50} values are most likely higher because at the range of concentrations tested, maximum response was not reached.

**FGF2 variant**	**thermal treatment**	**EC_{50} [nM]**	
		**no heparin**	**with heparin**
FGF2-wt	-20°C	0.120	NA
	37°C, 7 days	3.090 (!)	0.145
	37°C, 30 days	4.298 (!)	0.318
	50°C, 24 h	4.011 (!)	0.560
FGF2-STAB1	-20°C	0.024	NA
	37°C, 7 days	0.037	0.037
	37°C, 30 days	0.156	0.104
	50°C, 24 h	0.160	0.086
FGF2-STAB2	-20°C	0.015	NA
	37°C, 7 days	0.021	0.015
	37°C, 30 days	0.038	0.029
	50°C, 24 h	0.043	0.048
